# Translation and Validation Testing of the Constipation-Related Quality of Life Scale for Use in Japan

**DOI:** 10.7759/cureus.48661

**Published:** 2023-11-11

**Authors:** Sugihiro Hamaguchi, Madhulika G Varma, Hiroaki Nakagawa, Akihiro Ozaka, Sayaka Shimizu, Takako Maeshibu, Takafumi Wakita, Joseph Green, Shunichi Fukuhara

**Affiliations:** 1 General Internal Medicine, Fukushima Medical University, Fukushima, JPN; 2 Center for Innovative Research for Communities and Clinical Excellence (CiRC2LE), Fukushima Medical University, Fukushima, JPN; 3 Surgery, University of California, San Francisco, USA; 4 General Medicine, Shirakawa Satellite for Teaching and Research (STAR) Fukushima Medical University, Shirakawa, JPN; 5 Research Section, Patient Driven Academic League (PeDAL), Tokyo, JPN; 6 Community Medicine, Section of Clinical Epidemiology, Graduate School of Medicine, Kyoto University, Kyoto, JPN; 7 Graduate School of Psychology, Kansai University, Osaka, JPN; 8 Faculty of Sociology, Kansai University, Osaka, JPN; 9 Graduate School of Medicine, The University of Tokyo, Tokyo, JPN; 10 Health Policy and Management, Johns Hopkins Bloomberg School of Public Health (JHSPH), Baltimore, USA

**Keywords:** psychometric testing, translation and cultural adaptation, patient reported outcome measures, quality of life, constipation

## Abstract

Introduction

Establishing a scale that can easily be used to appropriately measure the impact of constipation on the quality of life in Japan is a first step toward addressing this important health issue. We developed a Japanese language version of the Constipation-Related Quality of Life scale, which has 18 items and four subscales, and then subjected it to validation testing.

Methods

After translation according to a standardized and commonly used procedure, the Japanese version of the Constipation-Related Quality of Life scale was administered to people in an internet-based panel, in March 2023. The participants included 1,276 adults who had constipation (median age: 60 years, 690 {54.1%} males). The outcome measures included the Constipation-Related Quality of Life scale, the Constipation Scoring System (an index of constipation severity), and the Medical Outcomes Study (MOS) eight-item short form (a measure of generic health-related quality of life).

Results

Confirmatory factor analysis (four-factor model) indicated that all 18 Constipation-Related Quality of Life items had sufficiently high factor loadings (0.686-0.926). Internal consistency reliability was high (Cronbach’s alpha: 0.86-0.94). Scores on the social impairment subscale and on the distress subscale of the Constipation-Related Quality of Life scale were significantly worse in the participants who had worse scores on the social functioning and mental health domains, respectively, of the MOS eight-item short form, which indicates good concurrent validity. Regarding criterion-based validity, the four subscale scores differed significantly among the four constipation-severity groups. The four subscale scores were also 1.16-4.53 times more sensitive than the MOS eight-item short form’s mental component score to differences among the four constipation-severity groups (relative validity: 1.16-4.53), which indicates good discriminant validity.

Conclusion

The Japanese version of the Constipation-Related Quality of Life scale can be used with confidence in its factor structure, its concurrent, criterion-based, and discriminant validity, and its internal consistency reliability.

## Introduction

The prevalence of constipation in the community worldwide is 14%, and it increases with age [1]. Constipation has been reported to be associated with impaired quality of life (QOL) [2], as well as lower labor productivity [3] and lower survival rates [4]. The impact of constipation on QOL is comparable to that of other chronic conditions (diabetes, inflammatory bowel disease, rheumatoid arthritis, coronary artery disease) [2].

Several constipation-related QOL measures have been developed [5-9]. The Constipation-Related Quality of Life (CRQOL) is a self-administered questionnaire consisting of 18 items and four domains (social impairment, distress, eating habits, and bathroom attitudes) that assesses the impact of constipation on the social, mental, and physical aspects of quality of life in terms. The CRQOL was developed and validated in the United States and its measurement properties are good with a relatively small number of questions [10]. This scale has not been validated in Japan.

Establishing a scale for easily and appropriately measuring the impact of constipation on QOL among Japanese people is a first step toward addressing this important health issue. Hence, this study aimed to translate the CRQOL into Japanese and investigate the psychometric properties of the Japanese version of the CRQOL.

## Materials and methods

Study design and setting

This study was conducted in the following two phases: (1) translation and cultural adaptation and (2) psychometric testing.

Phase 1: Translation and Cultural Adaptation

This phase was conducted from December 2022 to March 2023, based on the principles of good practice for the translation and cultural adaptation process of the International Society for Pharmacoeconomics and Outcomes Research (ISPOR) task force for translation and cultural adaptation [11]. First, we obtained permission from the original CRQOL developer to develop the Japanese version. The following process consisted of (1) forward translation, (2) reconciliation, (3) expert review, (4) cognitive debriefing, (5) review of cognitive debriefing results, (6) back translation, (7) back translation review and finalization, and (8) proofreading, with timing and participants shown in the figure in the appendix.

Forward translation: The original CRQOL was independently translated into Japanese by two general practitioners who are native Japanese-speaking speakers and fluent in English (HN, AO).

Reconciliation: The two independent forward translations were merged into a single translation through the discussion about the discrepancies with the forward translators (HN, AO) and a general practitioner who is also a native Japanese-speaking speaker and fluent in English (SH). 

Expert review: The forward translation after reconciliation was reviewed by the methodologists (TW and SF, expertise in scale development) with the forward translators (HN, AO, SH). This review ensured that the equivalence between the original and the translation was maintained, and necessary wording modifications were made to the forward translation.

Cognitive debriefing: Cognitive debriefing was conducted at an outpatient clinic of a hospital in Fukushima, Japan between January 23 and February 6, 2023. The participants were 10 patients who were aged 18 years and older, all of whom had constipation symptoms or were undergoing treatment for constipation. All were native speakers of Japanese and all agreed to participate in the study. Those with dementia or serious comorbidities were excluded. As an honorarium, participants were offered 1,000 yen. Each participant responded to the translated questionnaire that resulted from an expert review, and each was interviewed by the author (HN) according to the interview guide (the table in the appendix), primarily regarding comprehensibility.

Review of cognitive debriefing results: Four researchers (HN, AO, SH, SS) and two methodologists (TW, SF) reviewed the cognitive debriefing results to improve the wording of the translation.

Back translation: The forward translation after the modification from cognitive debriefing results was back-translated into English by a professional translator who is a native speaker of English, is fluent in Japanese, and has no prior knowledge of the CRQOL.

Back translation review and finalization: The back-translated version was then reviewed by the original developer of the CRQOL (MGV), and any discrepancies were discussed with four researchers (HN, AO, SH, SS), two methodologists (TW, SF), and a professional translator until a consensus was reached. 

Proofreading: The translation at this point was checked for typographic, and grammatical errors by HN, SS, and TW and was then used as the final version of the Japanese CRQOL.

Phase 2: Psychometric Testing

For psychometric testing, between March 22 and March 24, 2023, we conducted a cross-sectional survey of a panel of people who had constipation symptoms (provided by Cross Marketing Inc., https://www.cross-m.co.jp/) using an internet-based survey system (CREATIVE SURVEY Inc., https://jp.creativesurvey.com/). The present report complies with the consensus-based standards for the selection of health status measurement instruments (COSMIN) reporting guideline for studies on measurement properties of patient-reported outcome measures [12].

Participants: The participants comprised an internet-based panel of people in Japan who were 18 years of age or older, and who self-reported "having symptoms of constipation" (as per the regulation of Cross Marketing Inc.). The aim and outline of the study were presented at the beginning of the survey, and those who consented to participate responded to the questionnaire. Participants received an honorarium in accordance with the regulation of Cross Marketing Inc. Participants who had very short response times (below the 25th percentile, i.e., 6.9 minutes) and those who indicated that they had all comorbidities listed on the questionnaire (31 medical conditions) were excluded from the analysis because it is likely that they did not respond to the survey appropriately.

The sample size was planned to be sufficiently larger than the number of participants needed for factor analysis (i.e., seven times the number of CRQOL items {18 items}), and feasible in an internet-based panel survey [13].

Measurement: The final version of the Japanese CRQOL was used to measure constipation-specific QOL. The CRQOL has a recall period of one year. Each of its 18 items has five response options, which were scored 1-5. Four subscale scores were also computed as follows: social impairment (five items, score range: 5-25), distress (six items, score range: 6-30), eating habits (three items, score range: 3-15), and bathroom attitudes (four items, score range 4-20). Higher CRQOL scores indicate worse constipation-specific QOL. The Medical Outcomes Study (MOS) eight-item short form (SF-8) was used to measure generic QOL [14]. From the SF-8, the standardized mental component summary (MCS) and physical component summary (PCS) scores and the scores on the items measuring social functioning and mental health were computed, with higher SF-8 scores indicating better generic health-related QOL.

Constipation severity was measured by using the constipation scoring system (CSS) [15]. Questions on the CSS concern the frequency of bowel movements, painful evacuation efforts, feeling of incomplete evacuation, abdominal pain, minutes in lavatory per attempt, type of assistance, unsuccessful attempts at evacuation per 24 hours, and duration of constipation. Possible CSS scores range from 0 to 30, with higher scores indicating more severe symptoms.

For each participant, the following variables were also measured: age, gender, comorbidities, type of work (full-time, part-time, student, not working, and other), and frequency of outings (almost every day, four to five times a week, two to three times a week, about once a week, two to three times a month, and rarely).

Statistical analyses

Descriptive Analyses

For the sociodemographic characteristics, continuous variables are expressed as medians and interquartile ranges (IQR), while categorical variables are expressed as numbers and percentages.

*Psychometric Properties of the CRQOL* 

Factor structure: Based on prior studies, a four-factor model was hypothesized for the CRQOL [5]. Confirmatory factor analysis (CFA) was used to test this hypothesized four-factor model, assuming a correlation between the factors. Factor loadings (the extent to which a factor explains an item score) were computed and loadings greater than 0.4 were interpreted as substantial.

To evaluate the fit of the data to the CFA model, we computed the root mean square error of approximation [16] and the comparative fit index [17]. To examine whether the model fit was robust for different severities of constipation, these indices were calculated for each of the four groups that were defined by the CSS score quartiles. For the root mean square error of approximation, values generally considered to be acceptable are less than or equal to 0.10 [18], and for the comparative fit index, they are greater than or equal to 0.90 [19].

Internal consistency: We calculated Cronbach's alpha coefficient for each subscale of the CRQOL. To examine whether internal consistency was robust to different severities of constipation, alpha was calculated for each of the four groups that were defined by CSS score quartiles. Coefficient alpha can range from 0 to 1, with values greater than 0.7 considered to indicate reliability sufficient for group comparisons [20].

Hypothesis testing for concurrent, criterion-based, and discriminant validity is discussed below.

Hypothesis 1 (concurrent validity): We hypothesized that more social impairment due to constipation would be associated with worse QOL in the social domain. Specifically, we hypothesized that the CRQOL social impairment subscale score would be worse in the group that had lower scores on the SF-8 social functioning item.

Hypothesis 2 (concurrent validity): We hypothesized that more distress due to constipation would be associated with worse QOL in the mental health domain. Specifically, we hypothesized that the CRQOL distress subscale score would be worse in the group that had lower scores on the SF-8 mental health item.

Hypothesis 3 (criterion-based validity): We hypothesized that more severe constipation (an external criterion) would be associated with worse constipation-related QOL. Specifically, we hypothesized that the CRQOL scores would differ among constipation-severity groups defined by CSS scores and that the groups with higher CSS scores would have lower CRQOL scores.

Hypothesis 4 (discriminant validity): We hypothesized that for measuring how much QOL is affected by constipation severity, the CRQOL is more valid than the SF-8 MCS. Specifically, we hypothesized that the CRQOL is better than the SF-8 MCS for discriminating among constipation severity groups defined by CSS scores.

To test hypothesis 1, the CRQOL social impairment subscale scores were tabulated by groups defined by their response to the SF-8 social functioning item and subjected to analysis of variance (ANOVA). To test hypothesis 2, the CRQOL distress subscale scores were tabulated by groups defined by their response to the SF-8 mental health item and subjected to ANOVA. To test hypothesis 3, the subscale scores of the CRQOL were tabulated by CSS quartile-defined groups and subjected to ANOVA. To test hypothesis 4, SF-8 MCS and PCS scores were tabulated by CSS quartile-defined groups in addition to the subscale scores of the CRQOL and subjected to ANOVA. For each of the four CRQOL subscale scores, the RV was the ratio of the F statistic from the ANOVA for that subscale score to the F statistic from the ANOVA for the reference score, where the reference score was the SF-8 MCS score. That ratio was used to compare the validity of those two scores for discriminating across the groups differing in constipation severity. An RV greater than 1.0 indicates in proportional terms the extent to which the relevant score is more valid than the SF-8 MCS score, as in previous psychometric [21] and clinical studies [22].

R version 4.1.2 (Vienna, Austria: R Core Team, 2021) was used for the analyses. P-values that were less than 0.05 were taken to indicate statistical significance.

## Results

Participants’ characteristics

The cognitive debriefing had 10 participants. Of the 10, five were male and their median age was 60 (range: 45-90) years. All were taking at least one laxative (magnesium oxide in 10 and sennosides in one), and six of them had a history of stroke, five had a history of hypertension, two had a history of diabetes mellitus, and two had a history of chronic heart failure.

The characteristics of the participants in phase 2 (n=1,276) are shown in Table 1. Their median age was 60 years (IQR: 48.0-70.0), 690 (54.1%) were males, and 1,015 (79.5%) had one or more comorbidities. Laxatives, digital assistance, or enemas were used by 798 (62.5%) participants, and 1,195 (93.7%) participants had a history of constipation for one year or longer. The median CSS score was 10 (IQR: 7-14).

**Table 1:** Characteristics of participants in the internet-based panel survey that was used for psychometric testing. CSS: Constipation Scoring System

Variables			Total (n=1,276)	Percentages
Age (years), median age=60 (IQR: 48-70)	40		173	13.6%
	41-50		344	27.0%
	51-65		284	22.3%
	66-80		475	37.2%
Gender (male)			690	54.1%
Number of comorbidities		None	261	20.5%
		1-2	425	33.3%
		3-4	313	24.5%
		5	277	21.7%
Employment		Full time	454	35.6%
		Part time	183	14.3%
		Student	9	0.7%
		Others	630	49.4%
Frequency of outings		Almost every day	434	34.0%
		4-5 times a week	355	27.8%
		2-3 times a week	280	21.9%
		About once a week	99	7.8%
		2-3 times a month	47	3.7%
		Rarely	55	4.3%
		Missing	6	0.5%
Assistance with bowel movements		None	478	37.5%
		Laxatives	601	47.1%
		Digital assistance or enema	357	28.0%
Duration of constipation		<1 year	81	6.3%
		1-5 years	287	22.5%
		5-10 years	227	17.8%
		10-20 years	187	14.7%
		20 years or longer	494	38.7%
CSS score			10	IQR: 7-14

Factor structure

**Figure 1:**
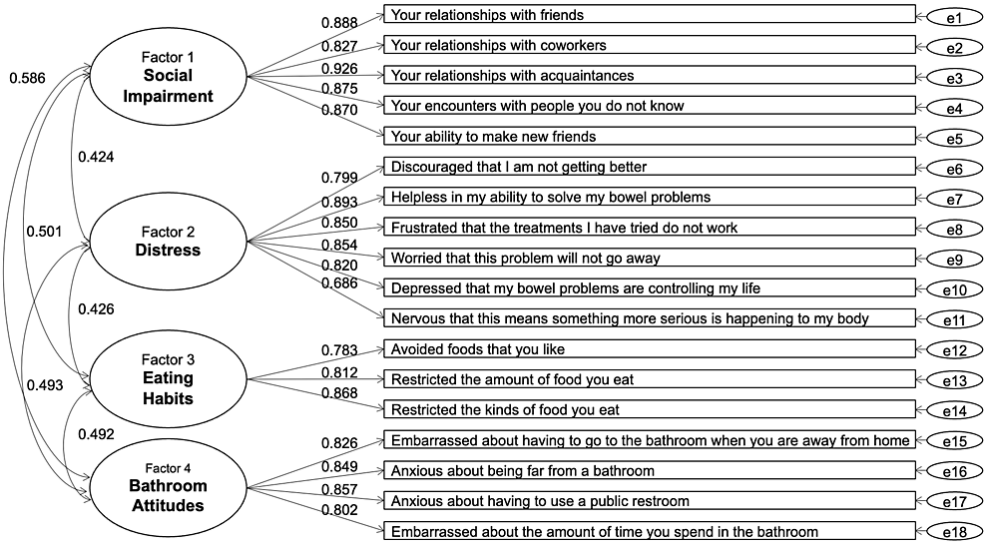
Path diagram showing factor loadings and correlations between factors from confirmatory factor analysis of CRQOL scores. CRQOL: Constipation-Related Quality of Life

The factor loadings and the correlations between the factors from the CFA (four-factor model) ranged from 0.686 to 0.926 and 0.424 to 0.586, respectively (Figure 1). The root mean square error of approximation and the comparative fit index were 0.08 and 0.94, respectively, and these indices of model fit were comparable in groups with different constipation severities (Table 2).

**Table 2:** Goodness of fit from confirmatory factor analysis of CRQOL scores and internal consistency of CRQOL subscales. ^a^Higher scores indicate more severe symptoms. CSS: Constipation Scoring System; CRQOL: Constipation-Related Quality of Life

Total (n=1,276)			Constipation severity (CSS^a^ quartile-defined groups)			
			0-7	8-10	11-14	15-30
			n=323	n=320	n=354	n=279
Goodness of fit (confirmatory factor analysis)	Root mean square error of approximation	0.08	0.08	0.09	0.09	0.10
	Comparative fit index	0.94	0.94	0.91	0.92	0.91
Internal consistency (Cronbach's alpha: 0.86-0.94)	Social impairment	0.94	0.97	0.90	0.93	0.94
	Distress	0.92	0.89	0.88	0.89	0.88
	Eating habits	0.86	0.79	0.81	0.82	0.88
	Bathroom attitudes	0.90	0.84	0.86	0.88	0.90

Internal consistency reliability

The computed values of Cronbach’s coefficient alpha ranged from 0.86 (eating habits) to 0.94 (social impairment). The values of alpha were similar in groups with different constipation severities (Table 2).

Concurrent validity: CRQOL social impairment subscale and SF-8 social functioning (hypothesis 1)

**Figure 2:**
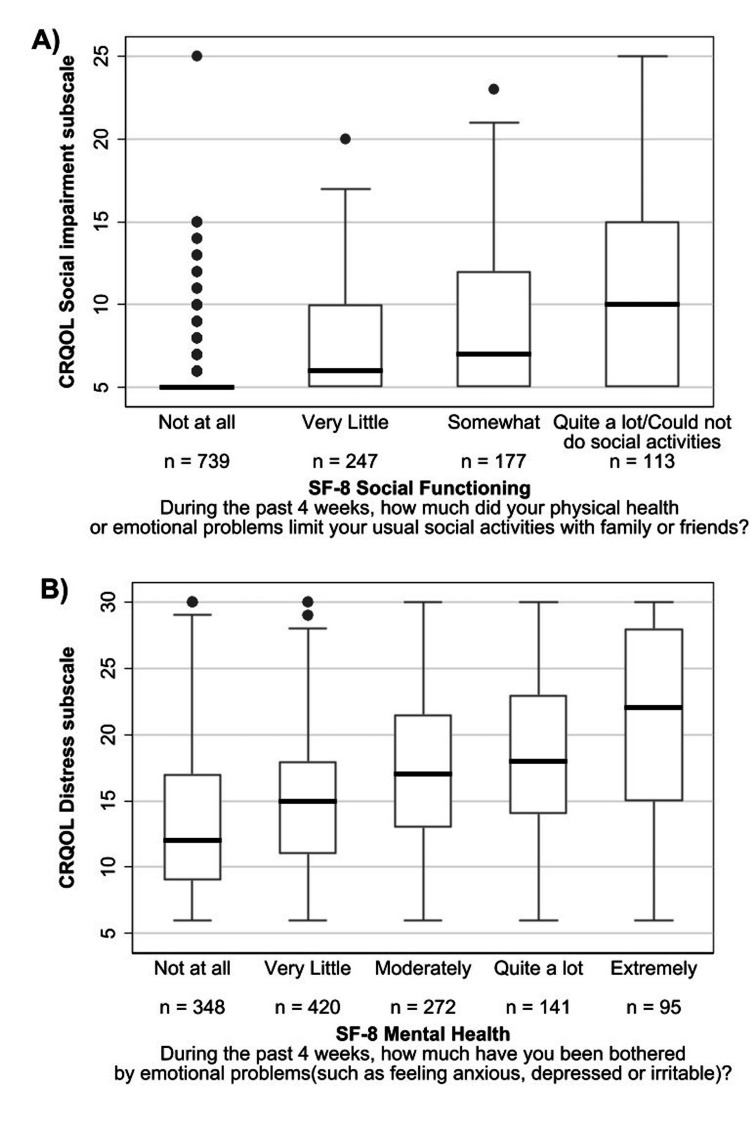
Concurrent validity of the CRQOL using the SF-8 (n=1,276). The images show (A) the CRQOL social impairment subscale scores for the groups defined by responses to the SF-8 social functioning item. Because the number of respondents in the group who responded “could not do social activities” on the SF-8 social functioning item was small (n=30), the group was combined with the group who responded “quite a lot” (n=83). (B) The CRQOL distress subscale scores for the groups defined by responses to the SF-8 mental health item. CRQOL: constipation-related quality of life

In this analysis, because the number of respondents in the group who responded “could not do social activities” on the SF-8 social functioning item was small (n=30), the group was combined with the group of those who responded “quite a lot” (n=83). The median scores on the CRQOL social impairment subscale were 5 (IQR: 5-5) for the “not at all” group, 6 (5-10) for the “very little” group, 7 (5-12) for the “somewhat” group, and 10 (5-15) for the “quite a lot/could not do social activities” group of SF-8 social functioning (F=133.70, p<0.001, η^2^=0.24 from ANOVA) (Figure 2, panel A).

Concurrent validity: CRQOL distress subscale and SF-8 mental health (hypothesis 2)

The median scores of the CRQOL distress subscale were 12 (IQR: 9-17) for the “not at all” group, 15 (IQR: 11-18) for the “very little” group, 17 (IQR: 13-21.5) for the “moderately” group, 18 (IQR: 14-23) for the “quite a lot” group, and 22 (IQR: 15-28) for the “extremely” group of SF-8 mental health (F=48.49, p<0.001, η^2^=0.13 from ANOVA) (Figure 2, panel B).

Criterion-based validity: association of CSS scores (external criterion) with CRQOL scores (hypothesis 3)

ANOVA to test this hypothesis showed that all four CRQOL subscale scores clearly differed as hypothesized between CSS quartile-defined groups (Table 3).

**Table 3:** Validity of the CRQOL for discriminating between constipation-severity groups compared with the SF-8 (n=1,276). ^a^Higher scores indicate better quality of life. ^b^Higher scores indicate worse quality of life. Values in square brackets indicate the possible range of the scores. ^c^Higher scores indicate more severe symptoms. ^d^F statistics, p-values, and η^2^ were computed through analysis of variance for each score by constipation severity groups. ^e^Each relative validity (RV) was computed by dividing the F statistic from the analysis of variance for the relevant score by the F statistic from the analysis of variance for the reference, which in this case was the SF-8 Mental Component Summary score. CSS: Constipation Scoring System; RV: relative validity; MCS: mental component summary; PCS: physical component summary; CRQOL: constipation-related quality of life Scores are presented as means (standard deviation).

Total (n=1,276)			Constipation severity (CSS^c ^quartile-defined groups)				F statistics^d^	p-Value^d^	η^2^	RV^e^
			0-7	8-10	11-14	15-30				
			n=323	n=320	n=354	n=279				
SF-8^a^	MCS (reference)	47.96 (7.53)	50.62 (6.11)	48.87 (6.61)	47.98 (7.19)	43.82 (8.63)	47.85	<0.001	0.10	1.00
	PCS	47.96 (6.81)	50.59 (5.19)	49.06 (5.99)	47.25 (6.59)	44.56 (7.98)	48.04	<0.001	0.10	1.00
CRQOL^b^	Social impairment 5-25	6.92 (3.55)	5.67 (2.12)	6.37 (2.48)	6.90 (3.22)	9.04 (5.06)	55.57	<0.001	0.12	1.16
	Distress 6-30	15.85 (6.11)	11.07 (4.25)	14.63 (4.73)	17.21 (5.29)	21.06 (5.56)	216.78	<0.001	0.39	4.53
	Eating habits 3-15	4.99 (2.49)	3.89 (1.57)	4.73 (2.12)	5.22 (2.36)	6.28 (3.19)	54.12	<0.001	0.11	1.13
	Bathroom attitudes 4-20	7.80 (4.04)	5.62 (2.35)	7.18 (3.28)	8.25 (3.90)	10.49 (4.82)	93.20	<0.001	0.18	1.95

Discriminant validity: comparison of association among different constipation severities, CRQOL vs. SF-8 (hypothesis 4)

Analysis to test hypothesis 4 showed that RVs were 1.16 for the social impairment subscale, 4.53 for the distress subscale, 1.13 for the eating habits subscale, and 1.95 for the bathroom attitudes subscale (Table 3).

## Discussion

A Japanese version of the CRQOL, a constipation-related quality of life scale, was developed using a standard method of translation and cultural adaptation. This Japanese version underwent psychometric validation testing using data from an internet-based panel of people who had constipation. The results were consistent with the hypothesized factor structure. The results of the concurrent, criterion-based, and discriminant validation tests were also as hypothesized, and internal consistency reliability was high. The establishment of the Japanese version of CRQOL scale will enable us to engage in international comparisons of research findings and international collaborative research about CRQOL using the same tool despite linguistic differences.

The CRQOL is not the only instrument available for measuring constipation-related quality of life. For example, the patient assessment of constipation quality of life (PAC-QOL) is a self-administered questionnaire consisting of 28 items in the following four domains: physical discomfort, psychosocial discomfort, worries/concerns, and satisfaction [6]. Versions of the PAC-QOL have been tested in several countries including Japan [23,24]. Nonetheless, the content validity of the PAC-QOL, i.e., whether it appropriately measures the concepts that it is intended to measure, remains questionable, particularly with regard to the "physical discomfort" domain [5]. In addition, “satisfaction” is usually considered to be separate from health-related quality of life [25]. Furthermore, the number of questions is large (28 items in the PAC-QOL vs. 18 items in the CRQOL), and we were interested in developing an instrument that would be less burdensome for respondents. Those considerations led us to use the CRQOL rather than the PAC-QOL.

Regarding factor structure, the results of the CFA (four-factor model) indicated that each item of the CRQOL had high factor loadings as in the original English version [5]. The goodness of fit of the model was also sufficiently high. Regarding concurrent validity, social functioning and mental health items of the SF-8 were associated with the CRQOL social impairment and distress subscales, respectively, as hypothesized.

Criterion-based validity of the CRQOL was also confirmed, with constipation severity measured by the CSS as the external clinical criterion. The CRQOL discriminated better between groups defined by constipation severity than did the SF-8 MCS. In particular, the sensitivity of the distress subscale to constipation severity was 4.53 times greater than that of the SF-8 MCS.

There are several limitations of this study. First, the original study also included healthy individuals for assessing discriminant validity. However, in our opinion, individuals with no constipation could not appropriately answer questions asking about the impact of constipation. In the present study, instead of including healthy individuals, we included a subgroup of participants who had a low score on the constipation severity scale, which, as the results show, was useful for quantifying discriminant validity. Second, the social impairment and eating habits subscales had substantial ceiling effects and relatively low discriminant validity. To reduce response burden, one option may be to offer a shorter version of the CRQOL that does not include the social impairment and eating habits subscales. Third, because this was a cross-sectional study, test-retest reliability and responsiveness were not assessed. Test-retest reliability was measured in the validation of the original English version, and it was found to be high [5]. Finally, we used an internet-based panel survey system for psychometric testing. Therefore, a selection bias may exist, and people with limited internet usage or low health consciousness may not have been included. However, we collected a large number of participants with constipation (1,276 participants), which may include patients with diverse backgrounds. Therefore, our participants may not be far from representing the Japanese population.

## Conclusions

The CRQOL was translated into Japanese according to a standardized and commonly used method. The factor structure of the Japanese version was consistent with the factor structure that was hypothesized a priori. In addition, the results of tests of concurrent, criterion-based, and discriminant validity were all good, and internal consistency reliability was high for all of the subscales. The Japanese version of the CRQOL can now be used with confidence, to measure the impact of constipation on QOL among Japanese people.

## Figures and Tables

**Figure 3:**
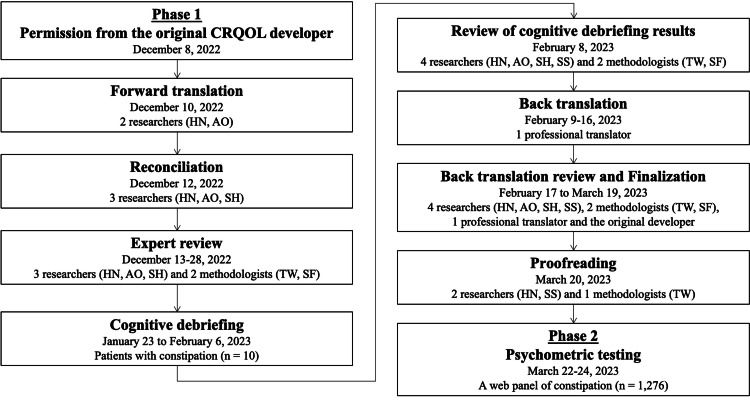
Steps in the translation (English to Japanese) and initial testing of the CRQOL instrument. CRQOL: Constipation-Related Quality of Life

## Appendices

**Table 4:** Interview guide used during cognitive debriefing after translation of the CRQOL instrument. CRQOL: Constipation-Related Quality of Life

	Questions
Instructions	Can you describe any confusion or difficulty you had in understanding these instructions? Are there any words or phrases that you would change to improve the instructions?
Recall	What period of time did you think about when you were completing the questionnaire? Did you always have the same degree of constipation or did it fluctuate from day to day or season to season?
Item stem	Were any of the questions difficult to understand? If so, please explain in your own words, please explain what you mean by this question.
Response options	Were there any options in the question that were difficult to understand? Were there any options in the questions that were difficult to answer? For each choice, please tell us what you meant. If you had to choose option X, what would your experience be?
Content coverage	What other experiences do you have with constipation that are not covered in this questionnaire?
Format	Observe how the respondent completed this portion of the questionnaire. What suggestions do you have for changing the questionnaire so it is easier to complete?
Length	Is the questionnaire too long? Too short?
